# Influence of Oral Health Care Systems on Future Career Environment of Dental Students in Europe

**DOI:** 10.3390/ijerph18168292

**Published:** 2021-08-05

**Authors:** Thomas Gerhard Wolf, Ernst-Jürgen Otterbach, Oliver Zeyer, Ralf Friedrich Wagner, Tin Crnić, Duygu Ilhan, Guglielmo Campus

**Affiliations:** 1 Department of Restorative, Preventive and Pediatric Dentistry, School of Dental Medicine, University of Bern, 3010 Bern, Switzerland; rwagner@kzvnr.de (R.F.W.); guglielmo.campus@zmk.unibe.ch (G.C.); 2 Department of Periodontology and Operative Dentistry, University Medical Center of the Johannes Gutenberg-University Mainz, 55131 Mainz, Germany; 3 FVDZ Free Association of German Dentists, 53177 Bonn, Germany; e-j.otterbach@fvdz.de (E.-J.O.); president@edsaweb.org (T.C.); 4 SSO Swiss Dental Association, 3000 Bern, Switzerland; oliver.zeyer@sso.ch; 5 Association of Statutory Health Insurance Dentists North Rhine (KZV Nordrhein), 40237 Düsseldorf, Germany; 6 EDSA European Dental Students’ Association, D02 PN40 Dublin, Ireland; 7 Periodontology Department, School of Dentistry, Istanbul Medipol University, Beykoz, İstanbul 34810, Turkey; duyguilhan@yahoo.com; 8 Turkish Dental Association (Türk Dişhekimleri Birliği), Ckurambar, Ankara 06530, Turkey; 9 Department of Surgery, Microsurgery and Medicine Sciences, School of Dentistry, University of Sassari, I-07100 Sassari, Italy; 10 School of Dentistry, Sechenov First Moscow State Medical University, 119435 Moscow, Russia

**Keywords:** future career, expectations, oral health care system, dental practice, Europe, dental students

## Abstract

Oral healthcare is organized subsidiarily and independently by nation states in Europe and also within the EU and consequently, major differences between the nation states and the various oral healthcare systems in Europe are present. The socialization in the respective catchment area can have an impact on the job choice and the perception of employment opportunities of different professional groups. Therefore, the purpose of this survey was to elucidate the influence of different oral healthcare systems on students living or studying in the respective catchment area. A questionnaire (in English, French, German, Italian, and Spanish) with 18 different components was administered. Data on gender, age, country of origin, university, semester, nationality, expected time of graduation, and forecast for future professional practices were gathered. In addition, 3851 students participated (2863 f/988 m). The sample distribution was uneven with predominantly Bismarckian and Southern European System participants. The National oral health care system was statistically significantly linked (*p* < 0.01) to the ownership period of a dental practice. Students in Bismarckian and Nordic systems tended to find their own practice earlier than in the Beverdigian system or Southern European and Transitional—East European systems. An association between the oral health care system and vocational training was inhomogeneous, but also significantly different (*p* < 0.01). The majority (47.51%, *n* = 1555) would like to work in their own practice, 18.95% (*n* = 621) want to establish a practice with two or more owners. It was striking that no student would like to work in the investor practice/practice chain of both Nordic, Beveridgian and Transitional—East European countries systems (*p* < 0.01). The oral health care system in which a dental student grows up/resides/studies influences the career choice/perception of future professional practice.

## 1. Introduction

European Union (EU) countries have the obligation to plan and provide health services and medical care to the citizens. European public health policies aim to protect and improve the overall health of EU citizens, advocate for the modernization of health infrastructure, and improve the efficiency of the health system. Moreover, the authorities involved in health at EU, national, regional, and local levels, as well as other stakeholders contribute. Health policies and care systems are still under the competence of the different EU countries [1]. However, the different national health systems and the provision of health services in EU countries, geographical Europe, and the WHO European region are quite diverse [2,3].

Key issues are the so-called “four freedoms” in the EU single market: Freedom of movement for people, assets, capital, and services. [4]. Within the framework of the freedom of movement of persons, all EU citizens enjoy the right to reside, exercise a profession, and remain in any country of the EU. Since 1992, self-employed professionals are allowed to work in any EU country, “the so-called freedom of establishment”, meaning the liberalization of the provision of services, such as consulting, insurance, and other services. The flow of capital between member states is not subject to any restrictions within the framework of the free movement of capital [4]. The movement of money, capital, and payments in the EU has been fully liberalized by the European Economic and Monetary Union and increasingly coordinated by fiscal and monetary policy. Therefore, the restrictions on the movement of capital between the national EU member states and third countries should be largely eliminated. The free movement of capital should underpin the internal market and thus promote economic growth, to which the creation of an international currency (€/EURO) should also contribute [5]. Health policy in particular is subject to special regulations. Politically created reform processes are intended in many places to increase quality and at the same time reduce costs. However, changes in the healthcare market are dependent on numerous conditions [6], and the healthcare market can thus be described as a sensitive area of liberal service provision [7].

The systems of financing and organizing oral health care in the EU/EEA countries have their own national, historical, political, and socio-economic traditions [2,8]. There are several considerations for using indicators to conduct comparative analyses between oral health systems of countries [9,10]. While the Beveridgian (national health care system) and Bismarckian (social insurance system) health care systems can be very clearly distinguished from each other, there are also numerous hybrids of the two systems in Europe [2,8]. While the financing of the health care system in the Bismarckian model functions by means of compulsory contributions from employers and employees to the social security system, the health care system in the Beveridgian model is financed by public taxes and thus the facilities are financed directly by the state [8].

Mixed health care systems are often characterized by private financing through voluntary insurance or even prepayment. In the last decade, a change in the oral health care service provision can be observed [7,11,12,13,14]. While fewer dentists in the European region want to start their own practice—still the most common form of dental professional practice in Europe [7,15]—than in the past, the number of employed dentists is increasing and especially of larger practice structures and dental chains, which are managed by non-dentally trained directors and often used as a profit investment by financially strong investors [16,17]. It is not yet possible to foresee what developments will result from this change in the dental care landscape in numerous European countries.

However, it is necessary to examine the extent to which the career choices of dentists will change in the future in order to provide assistance to policy makers in setting a course in good time.

Therefore, the influences of the oral health care systems will be investigated in this study towards the career choice of dental students. Our null hypothesis was that the career prospects of young European dental students are independent of the oral health systems of the different countries. To validate this hypothesis, a multicenter online cross-sectional survey was designed, planned, and conducted.

## 2. Materials and Methods

The European Regional Organization of the FDI World Dental Federation (ERO-FDI) consists of member associations from 37 of 53 national states of the WHO European Region [18]. While the population of the 27 European Union member states in 2020 was around half of the WHO European Region, at ~448 million people (~218 million males and ~229 million females) [19], the WHO European region has a population twice as high than the European Union of almost 900 million people [20]. According to a recent study of Poutvaara et al. (2019) [21], the principles of free movement of goods, services, and legislation in the European Union area generate benefits estimated at 386 billion € for free movements of goods and custom union at 189 billion € annually.

A power analysis and a sample size calculation were conducted before the survey. In the WHO European Region, 97,000 persons were attending in undergraduate dental schools in the academic year 2018/2019 [18] of which 16,166 students were in the last semester. The sample size was calculated using the online sampling calculator GLIMMPSE (General Linear Mixed Model Power and Sample Size https://glimmpse.samplesizeshop.org (accessed on 5 February 2020), with a hypothesized frequency of outcome factor in the population fixed at 50% with a variance of ±5 with a confidence level of 99.9%. The sample size was set at 1015 subjects.

### 2.1. Questionnaire

In the current survey conducted between November 2019 and January 2020, dental students of 40 different nationalities studying in the ERO-FDI countries (WHO Europe Region) filled in a self-administered online questionnaire in five languages: English, French, German, Italian, and Spanish. The national dental associations of the ERO-FDI forwarded the survey to their universities. The following organizations supported the study: ERO-FDI (European Regional Organization of the FDI World Dental Federation, Bern/Geneva, Switzerland), the IADS (International Association of Dental Students, Geneva, Switzerland), the FVDZ (Freier Verband Deutscher Zahnärzte; Free Association of German Dentists, Bonn, Germany), and the EDSA (European Dental Students’ Association, Dublin, Ireland). A short information leaflet explaining the aim of the survey and the questionnaire link was sent by e-mail to the dental students.

The questionnaire was submitted only to students of the last two semesters. An online question tool was used for all questionnaire items with answer categories directly on the mobile phone, computer or tablet (https://www.survey.consulimus.de/english/umfragen/dental-students-in-europe.html accessed between 1 September 2019 and 30 April 2020). The participation was on a voluntary and anonymous basis. All the procedures were in agreement with the ethical standards of the local research committee and the 1964 Declaration of Helsinki and its later amendments or comparable ethical standards. The approval of an ethical committee to collect and analyze anonymous data was not required following the Swiss law on human research (Humanforschungsgesetz, HFG). An informed consent was gained from all participants in the survey by accessing the online survey. The questionnaire was designed following similar questionnaires present in the literature [22,23,24,25,26] and was piloted by members of the ERO-FDI working group and several dental students who were invited to the working group meetings. Personal data on age, country of residence, nationality, university, current semester, expected year of graduation, compulsory postgraduate (professional) training after graduation, initial career plans after professional training, ideal type of study and reasons for this, expected time to be self-employed, influence of family life on career, reasons for working as an employed dentist and settings of an employed dentist, type of study and sex ratio were requested. Data from this study on the expectations of European students regarding their professional dental practice of this dataset have already been published [27]. The checklist for reporting results of internet e-survey (CHERRIES) has been used (Supplementary Materials) [28].

### 2.2. Health Care Systems—Classification

The health care system classification was derived from the paper of Nikolovska [8]. The countries were divided into five main groups: Nordic countries, Bismarckian System, Beveridgian System, Southern European System, and Transitional—East European countries. The countries were combined as follows to perform the statistical calculation: Nordic countries: Finland, Norway, Sweden; Bismarckian System: Austria, Belgium, France, Germany, Luxembourg, Switzerland; Beveridgian System: United Kingdom; Southern European System: Italy, Greece, Portugal, Spain, Turkey; and Transitional—East European countries: Czech Republic, Estonia, Hungary, Latvia, Lithuania, Poland, Slovakia, Slovenia.

### 2.3. Data Analysis

The survey replies were entered into ExcelTM (Microsoft Corp., Redmont, WA, USA) 2019 for Macintosh (Apple Inc., Cupertino, CA, USA). For the statistical evaluation, the data were cleaned and then transferred to STATA16^TM^ (StataCorp LLC, College Station, TX, USA) for their statistical analysis. Questionnaires with less than five questions completed (<25%) were removed from the final dataset and not used for the survey (*n* = 129). For each entry, the relative and absolute frequencies were determined. If one cell had a value of less than five, the difference in proportion was assessed with the χ^2^ test or the Fisher exact test. *Post hoc* estimation was evaluated using multiple tests such as the expected and observed number of frequencies, the χ^2^ contribution, and the proportion. The Likert scale was used to construct some items [29]. The significance level was set at *p* < 0.05.

## 3. Results

**Figure 1:**
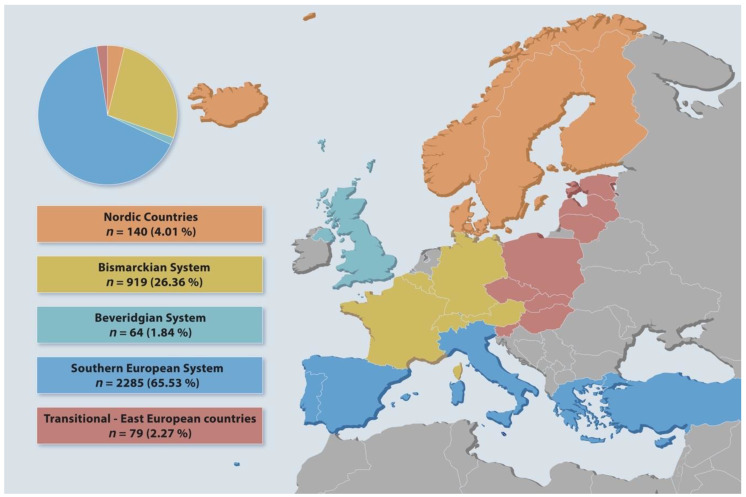
Map of Europe with information on the type of health care systems of the study participants.

The questionnaire was filled in by 3851 subjects, 988 males and 2863 females. The sex ratio (M/F) was 0.34 with a no-response rate of 3.50% (*n* = 129). A map of Europe with information on the type of health care systems of the study participants is shown in Figure 1.

### 3.1. Sample Distribution

The participants were classified according to the respective health system into Nordic countries, Bismarckian System, Beveridgian System, Southern European System, and Transitional—East European countries (Table 1). The study participants were mainly from Italy and Germany, so the distribution is predominantly towards Bismarckian and the Southern European System.

### 3.2. Association between Oral Health Care System and Planning Period to Own a Dental Practice

The National oral health care system was statistically significantly linked to the owner-ship period of a dental practice (χ^2^_(12)_ = 933.84 *p* < 0.01). The majority of the respondents (38.01%) would like to opt to have their own practice more than 3 years after the training period (vocational training) (Table 2), with approximately one third of the study participants wanting to spend either 1 or 2 (30.69%) or 3 years (31.29%) to establish their own practice.

**Figure 2:**
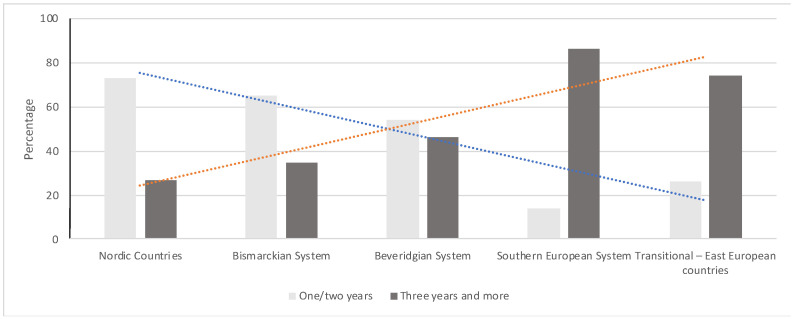
Distribution of the sample by the oral health care system and planning period to own a practice (grouped by 1/2 years or 3 years or more).

In Bismarckian systems, the study participants are conspicuously already in the first or second year for a foundation of their own dental practice, while in southern European systems, study participants are indicated as predominantly 3 or even more than 3 years (Figure 2 with trend lines in blue and red). The Nordic countries show a similar trend as the Bismarckian Systems, whereas, Transitional—East European System countries show a similar trend as the southern European countries. Only participants in the Beveridgian System indicate a desire to establish their own practice mainly after 2 and 3 years and less after 1 or more than 3 years.

### 3.3. Association between the Oral Health Care System and Vocational Training

The association between the oral health care system and vocational training is depicted in Table 3. The data are very dis-homogeneous, but the differences are significantly different (*p* < 0.01). While the overwhelming majority (70.55%) of the survey participants completed 1 year (70.55%) of vocational training, only a quarter of the study participants (24.90%), 4.55% stated that there would be no such vocational training.

### 3.4. Work Environment Where a New Dentist Would like to Work

The association between the type of institution where a new dentist would like to work and the oral health care system is shown in Table 4. Almost half of all study participants (47.51%) would like to work in their own practice, although this was the most frequently stated form of practice across all the systems, except for Nordic countries. A practice with two or more owners would like to establish 18.97% of the participants, but no participant in Transitional—East European countries. In addition, 13.60% choose an outpatient care center and 10.60% the national health system. The investor practice/practice chain is the least popular of all the systems (9.32%), with Southern European Systems (7.73%) and Bismarckian System (1.59%) being the most popular. Of the Nordic countries, the Beveridgian system and Transitional—East European countries choosing the investor practice/practice chain was not indicated by any participant (0.00%) (*p* < 0.01).

## 4. Discussion

A thorough search of the relevant literature yielded that this is the first survey aimed to investigate and address the influence of oral health systems in Europe on dental students’ career choices. In order to generate information on the design but also control of oral health care and to accompany the change in the dental field with its multiple challenges in Europe, an online survey was designed. Findings on the impact of the different oral health care systems in Europe on the future professional choice of dental students should be collected. The outcomes could be extremely relevant for policy makers and institutions, companies, and stakeholders involved in health care provision. It was found that the oral health care system in which a dental student grows up/resides/studies significantly influences career choice in terms of type of practice.

### 4.1. Limitations

The distribution of sex and nationality in the present survey is highly skewed. A higher percentage of female dental students participated in the survey, and this confirms the sex trend about dental profession in many European countries in recent years [7,15,16,30]. The dentistry profession has feminized less quickly than other professions (i.e., pharmacy, law, and medicine), but it has experienced a greater influx of women than other professions [31].

As various national dental organizations and contact persons did not find adequate contacts to support the survey at their university to the students, the majority were from Italy and Germany. However, even with this constraint, the overall sample size is quite large compared to studies among dental students [22,32]. It must also be pointed out that a certain bias is present simply due to the difference in knowledge among the students, since many students have not really dealt with the topic of future career choice or establishment of an own dental practice due to an early or preclinical semester or a lack of previous knowledge by a family member working in the dental profession. In a study as this, aiming to reach a percentage of the universe of dental students in each country, participation becomes a tough issue. This fact is supported by the high number of non-responders.

Another limitation might be that the questionnaire was submitted only in five languages, even if they accounted for more than 75% of the European population. This might have created some bias in the participation rate. Moreover, the circumstance that the current survey was conducted before the COVID-19 pandemic might be considered a serious limitation on the possibility to generalize the outcomes. The parameters included in the analysis should be re-examined taking into account the effect of the pandemic. The COVID-19 outbreak had a significant impact on public health [33,34]. Personal risk was increased, job security was suddenly questioned, and the high demands of the workplace could also change people’s interest in a career in health care [35].

### 4.2. Influence and Motives on the Choice of Profession

As far as the authors know, the impact of oral health care systems has not been studied. Therefore, no data are available for dentistry, nor for other health care professions. Motivations for studying dentistry vary widely. The motivations for studying dentistry ranged from the desire to be capable of helping others [36,37], achieving self-esteem and interests related to health care, but also the chance to have a well-paid prestigious position are reasons for choosing the dental profession [36,37]. Moreover, personal and family background, work-life balance [38], professional/social status [39,40], job security, flexible work hours [39], professional independence [37], intellectual challenges, security, recommendation by others, and non-admission to other degree programs [37] play a role in the choice, with data on sex, ethnicity, mode of entry into college [41], parental education, and marital status [37]. Some underlying factors also influenced the career choice as professional work environment, health care, etc. Taking into account the high unemployment of graduates in the public and private sector in several European countries, this may also play a crucial influencing factor [40]. In comparison to medical students, dental students displayed more professional attitudes, in which selflessness and intellectual defiance could be the key motivating factors. Dental students exhibited a stronger striving for financial and personal gain [42]. The association between the socio-economic status of parents and their offspring has emerged as one of the stylized facts in economics, although the variation between countries and occupations is substantial. There is evidence on the role of economic benefits that individuals obtain as adults due to their parents’ occupational status [43,44] suggesting a certain propensity for children to follow their parents’ career routes. It has already been confirmed that children of parents who work in dentistry are more likely to choose to study dentistry [22,23,27]. In this context, it is also necessary to underline that the cost or tuition of dental school has increased significantly, leading to potential student debt that must be financed largely by private loans for costs sometimes exceeding USD 25,000/year. The keenness of health profession courses for students to work in the countryside after graduation was reported [45]. It is also worth noting that female students and students who come from a rural community are also more inclined to opt for a career in rural areas [46]. A very good knowledge of one or more languages, a desire to relocate/migrate after graduation is a factor to be considered and taken into account [46]. Although a limited number of studies to date have examined career sex differences among young dentists with regards to career breaks, females are more likely than males to take career breaks early in their careers and to choose part-time work on their return [31,47]. Moreover, sex gaps should be considered to ensure adequate staffing in dentistry in the future [47].

## 5. Conclusions

Some considerations about the impact of oral health care systems on the attitude of European dental students for their future career choice can be drawn:

- The oral health care system in which a dental student grows up or resides or studies significantly influences career choice in terms of type of practice.
- Study participants from Nordic and Bismarckian systems would like to decide to start their own practice strikingly early in their first or second year.
- Nearly half of all the study participants would like to work in their own practice, and this was the most commonly reported practice type across all the systems except Nordic countries.
- A practice with two or more owners is what several participants would like to establish, but no one in the Transitional—East European countries would do so.
- An ambulatory care center or national health system are the practice of choice of just over a tenth of the survey participants.

## Figures and Tables

**Table 1:** Distribution of the sample by oral health care systems.

Oral Health Care Systems	Females *n (%)*	Males *n (%)*	Total *n (%)*
Nordic Countries	82 (2.35)	58 (1.66)	140 (4.01)
Bismarckian System	611 (17.52)	308 (8.83)	919 (26.36)
Beveridgian System	43 (1.23)	21 (0.60)	64 (1.84)
Southern European System	1415 (40.58)	870 (24.95)	2285 (65.53)
Transitional—East European countries	51 (1.46)	28 (0.80)	79 (2.27)

Non responders 56 χ^2^_(10)_ = 20.82 *p* < 0.01 not classifiable 308.

**Table 2:** Association between the oral health care system and planning period to own a practice.

	Nordic Countries *n* (%)	Bismarckian System *n* (%)	Beveridgian System *n* (%)	Southern European System *n* (%)	Transitional—East European Countries *n* (%)	Total *n* (%)
One year	53 (38.41)	248 (30.10)	13 (23.21)	168 (8.06)	6 (9.23)	485 (15.30)
Two years	48 (34.78)	290 (35.19)	17 (30.56)	122 (5.84)	11 (16.92)	488 (15.39)
Three years	22 (15.94)	71 (8.62)	17 (30.56)	872 (41.78)	10 (15.38)	992 (31.29)
More than three years	15 (10.87)	215 (26.09)	9 (16.07)	925 (44.32)	38 (58.47)	1205 (38.01)
Total	138 (4.35)	824 (25.99)	56 (1.77)	2087 (65.84)	65 (2.05)	3170 (100.00)

Non-responders/not classifiable *n* 681 χ^2^_(12)_ = 933.84 *p* < 0.01. Cramer’s V = 0.31.

**Table 3:** Association between the oral health care system and vocational training.

	Nordic Countries *n* (%)	Bismarkian System *n* (%)	Beveridgian System *n* (%)	Southern European System *n* (%)	Transitional—East European Countries *n* (%)	Total *n* (%)
Not exist	0 (0.00)	18 (1.96)	0 (0.00)	88 (4.22)	43 (66.15)	149 (4.55)
one year	91 (65.00)	528 (57.45)	44 (68.75)	1625 (77.94)	21 (32.31)	2309 (70.55)
Two or more years	49 (35.00)	373 (40.59)	20 (31.25)	372 (17.84)	1 (1.54)	815 (24.90)
Total	140 (4.28)	919 (28.08)	64 (1.96)	2085 (63.70)	65 (1.99)	3273 (100.00)

Non-responders/not classifiable *n* 578 Fisher’s exact test 3714.86 *p* < 0.01. Cramer’s V = 0.34.

**Table 4:** Type of institution where a new dentist would like to work.

	Nordic Countries *n* (%)	Bismarkian System *n* (%)	Beveridgian System *n* (%)	Southern European System *n* (%)	Transitional—East European Countries *n* (%)	Total *n* (%)
Individual practice	21 (0.64)	400 (12.22)	35 (1.07)	1065 (32.54)	34 (1.04)	1555 (47.51)
Practice with two or more owners	32 (0.98)	118 (3.61)	28 (0.86)	443 (13.53)	0 (0.00)	621 (18.97)
Investor practice/practice chain	0 (0.00)	52 (1.59)	0 (0.00)	253 (7.73)	0 (0.00)	305 (9.32)
Outpatient care center	44 (1.34)	165 (5.04)	0 (0.00)	216 (6.60)	20 (0.61)	445 (13.60)
National Health system	43 (1.31)	184 (5.62)	1 (0.03)	108 (3.30)	11 (0.34)	347 (10.60)
Total	140 (4.28)	919 (28.08)	64 (1.96)	2085 (63.70)	65 (1.99)	3273 (100.00)

Non-responders/not classifiable *n* 578 Fisher’s exact test 434.86 *p* < 0.01. Cramer’s V = 0.18.

## Data Availability

The data presented in this study are available on reasonable request from the corresponding author. The data are not publicly available due to the European General Data Protection Regulation GDPR.

## Supplementary Materials

The following are available online at https://www.mdpi.com/article/10.3390/ijerph18168292/s1, Checklist for Reporting Results of Internet E-Surveys (CHERRIES).
